# Motivational Factors in the Use of Videoconferences to Carry out Tutorials in Spanish Universities in the Post-Pandemic Period

**DOI:** 10.3390/ijerph181910474

**Published:** 2021-10-05

**Authors:** Alfonso Infante-Moro, Juan C. Infante-Moro, Julia Gallardo-Pérez, Antonio Luque-de la Rosa

**Affiliations:** 1 Department of Financial Economics, Accounting and Operations Management, University of Huelva, 21071 Huelva, Spain; juancarlos.infante@decd.uhu.es (J.C.I.-M.); julia.gallardo@decd.uhu.es (J.G.-P.); 2 Department of Education, University of Almeria, 04120 Almería, Spain; aluque@ual.es

**Keywords:** digital tools, teaching, motivational factors, ICT, education, COVID-19

## Abstract

Many of the tools used for virtual teaching during the pandemic had not been used previously, but they could continue to be used when traditional teaching returns. For this reason, this study focused on locating the key motivational factors for the possible continuation of the use of one of these tools, videoconferencing, to carry out tutorials in Spanish universities as a complement to face-to-face tutorials. For this, a literary review was conducted to obtain a list of motivational factors that may influence teachers to continuing using it, and a causal study was performed with university professors (through fuzzy cognitive maps) to identify the causal relationships among these factors and classify them by their relevance in making a decision. The most influential factors are intention, attitude and perceived compatibility with how tutorials are given, and the negative factors include quality management and trust.

## 1. Introduction

The COVID-19 pandemic brought about forced virtual teaching due to government-imposed mobility restrictions [1], which led to the promotion and use of technological tools that many teachers had never thought of incorporating into their teaching [2,3,4,5].

In Spanish universities, one such tool is videoconferencing to carry out virtual tutorials. It proved to be very useful and could continue to complement face-to-face tutorials once the pandemic ends. Any decision to continue videoconferencing will be made by teachers because they are the ones who will have to insert it into to their tutorial delivery; therefore, this study aims to identify the key motivational factors for teacher acceptance and help universities to encourage teachers to continue using it.

A literary review revealed a list of motivational factors for accepting an innovation or new teaching tool. A causal map was developed in which university professors at Spanish universities and experts in technological implementation evaluated and analyzed the most influential factors for teachers to consider. In collecting data to construct the map, a series of interviews was conducted, and a causal map was made for each interviewee. Later, a global map was made from the average assessments of the experts and analyzed through the FCMappers tool, which identified the most influential factors.

The following section contextualized the use of technological tools in education and their importance to teaching during the pandemic and detailed the motivational factors that influenced their acceptance. Then, we present the methodology, the analysis of our results, and our conclusions about the determining motivational factors.

## 2. Literature Review

The incorporation of electronic devices is at a high level in all areas of society [6,7,8,9,10,11], including education [12,13,14,15,16,17,18], both in their number and in their use by citizens [19,20,21,22,23,24]. Teaching is one area where great technological advances have been made, and these tools were already in use before the pandemic: massive open online courses (MOOCs) [25,26], online social networks [27,28,29], multimedia tools [30,31], virtual reality [32], robotics [33] and mobile phones [34,35,36,37]. Teachers involved in this study comprise both those who have used them and those who have not [38,39,40].

The use of each tool has specific motivational factors, but this study focused on the factors influencing teachers to use videoconferencing to carry out tutorials.

After a search of the main scientific databases (Web of Science, Scopus and Google Scholar) and after consulting articles, such as those by Sánchez-Prieto [41] and González-González [42], we opted for the factors in the study by R. Arteaga [43], which, through the partial least squares (PLS) methodology (based on expert judgment) and a previous literary review, pointed out motivational factors that influenced the use of an innovative tool:

- Quality Management (QM): the user’s degree of satisfaction based on the perceived quality of use [44] and measurable results [44,45,46,47]. Output quality focuses on user satisfaction based on whether or not the new technology performed its functions in an optimal way [44]. The demonstrable results focus on the user’s perception of tangible results, whether the expected results were achieved [45,46,47].
- Available Information (AI): the user’s degree of adoption and use based on existing information [48]. This factor is reflected in the user’s prior knowledge of the new technology, previous experience in its use, awareness of its existence, and degree of exposure to it.
- External Conditioning (EC): the influence of the environment on potential users, whether favorable or unfavorable [49,50,51]. This factor is reflected in social influence and facilitating conditions. Social influence focuses on the influences a user receives from other people [49] to use it, and the facilitating conditions focus on the organizational and technical infrastructures that support the use of this new technology [50,51].
- Trust (T): the user’s expected degree of security and privacy [52,53,54]. Security focuses on the user’s feeling of the absence of danger or risk when using the new technology [53,54], and privacy focuses on the feeling that the information given when using it is controlled and protected [53].
- Perceived Compatibility (PC): the degree of compatibility perceived by the user [46,55,56]. This factor is reflected in the connection between the new technology and the user’s needs, habits and desires [46,55].
- Perceived Usefulness (PU): the increase in the user’s own performance [57,58,59,60,61]. This factor depends on whether using the new technology helps the user complete a task and whether its use provided advantages over a traditional method [59,60,61].
- Attitude (A): the user’s perception of its advantages or disadvantages [62,63,64].
- Intention (I): elements that motivate the user to use a new technology [63,64].

The relationship of each of these to the use of a new technological tool can be seen in the studies cited, and the relationship of each of them with their corresponding factors was ratified in the study by R. Arteaga [43] through the PLS methodology based on the judgment of experts.

However, in the area of videoconferencing, no research has been conducted on its use in delivering teaching tutorials. Most studies on videoconferencing in teaching deal with educational experiences and the advantages and disadvantages of its use [65,66]. The most similar study assessed the degree of influence of each motivational factor independently without taking into account that the factors could influence each other [67,68], which could change the relevance of each of the factors in making decisions.

## 3. Methodology

Once the motivational factors were ascertained, the next step was to find out the factors underlying teachers’ decisions.

For this, the knowledge of experts was required and one of the most used methodologies in the study of knowledge was used, Fuzzy Cognitive Maps (FCMs) [69]. It provides visualization of factors that are part of a system or decision and the causal relationships between them [70,71]. The result allows the validation or exclusion of factors within that system or decision, and an explanation for that system or decision. Furthermore, if used together with software such as FCMappers, it helps make decisions and proposes possible strategies to follow [72,73].

Thus, because of this methodology, the motivational factors in a system to be studied, the existing influences among them and the degree of these influences were located.

The knowledge of the experts was obtained through an interview with each of them, in which they gave their opinions on the motivational factors that would influence teachers to accept videoconferencing. In each of these interviews, as in other studies that use fuzzy cognitive maps (for example, the study by J. Solana [74]), the interviewee was given two tables.

The first was a guide table with the motivational factors that influence users when using a new technological tool (obtained in the literary review), which served as support to the interviewees when defining the factors that influence this system and the causal relationships among them, although they had the possibility of adding some other factor if they thought it appropriate or eliminating any of those in the table (Table 1). 

The other table had a list of possible degrees of causal relationships between the factors identified within the system, which helped the interviewees assess these causal relationships (Table 2). In this table, in addition, these evaluations were accompanied by a numerical equivalent to be incorporated into the adjacency matrix that had to be filled in by each of the interviewees, a necessary conversion to be able to carry out the analysis with the FCMappers Software [75,76,77].

The adjacency matrix is a square matrix that is used in this type of study as a form of representation of cause–effect relationships among the factors, relationships that must be based on the opinion of each of the experts and that must be quantified by values of the interval (−1, 1) [75,76].

In this way, each interviewee had to establish the existence or non-existence of casual relationships that they detected in the system to be studied, and for this, they had to use the possible degrees of existence of causal relationships collected in Table 2, which later had to be converted into numbers in the interval (−1, 1) based on the numerical equivalences in the table [75,76].

With each interview, a representation of the adjacency system, or matrix, was obtained from the opinion of each of the interviewees, subsequently making a global representation or global adjacency matrix with the average of these results.

This set of results (global adjacency matrix) was analyzed through FCMappers Software [77], which classified all the variables that made up the system according to the level of influence with the rest of the variables, thereby giving the indicators “outdegree”, “indegree” and “centrality” [78].

The outdegree indicator marked the degree of influence exerted by one factor on the others; the indegree indicator marked the degree of influence that a factor received from the others; and the “centrality” indicator marked the total degree of participation of a factor in the system to be studied [78]. If one factor exerted a lot of influence, it had a high outdegree. If it was heavily influenced by others, it had a high indegree; and if the factor exerted and received many influences, it had a high centrality because this indicator is the sum of the outdegree and indegree indicators.

The number of interviewees in a study that uses a fuzzy cognitive map is fixed according to the number of new influencing factors per interview [79,80]; that is, the number of interviewees is adequate from the moment the interviewees stop providing new factors (to those provided by previous interviewees) to the system. This means that there was no predetermined number of interviewees, and that studies could have varying sample sizes: 45 [81], 41 [82], 30 [83,84], 29 [81], 8 [85,86] or 7 [74] and up to 4 experts [87]. In this study, the number of interviewees was set at 40 to give the results the greatest possible significance despite the fact that none added new factors, and this number could have been lower. In addition, no great differences were observed between the values contributed by each of the interviewees, which increased this significance.

These experts were university professors who responded to an invitation to participate in the research. They had training in Information and Communication Technologies (ICTs) and knowledge of how to apply them in an effective way for students. They have taught face-to-face or virtually at Spanish universities for more than 10 years and have a doctorate that includes research into information and communication technologies in teaching, online teaching and educational institutions. The majority (80%) were teachers of educational technology subjects in education.

## 4. Results

As stated in the previous section, each interview generated an adjacency matrix with information provided by each expert, and the average of the results generated the global adjacency matrix that was entered into the FCMappers software, which classified the factors of the system based on outdegree, indegree and centrality indicators.

The global adjacency matrix comprised 8 motivational factors and 54 cause–effect connections between them (Table 3).

**Figure 1:**
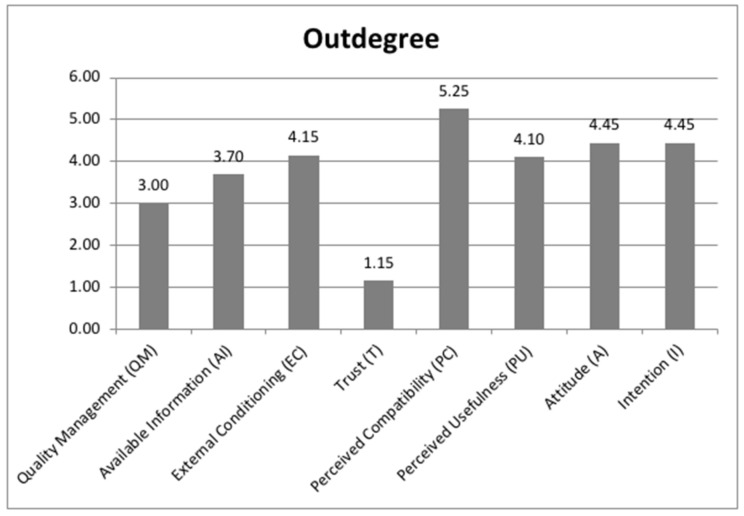
Indicators of the fuzzy cognitive map: outdegree.

After introducing this matrix into the FCMappers software, the factors with the highest outdegree, indegree and centrality indices were the following (Table 4):

Regarding the outdegree indicator, the classification of these factors from highest to lowest was perceived compatibility (PC), intention (I), attitude (A), external conditioning (EC), perceived usefulness (PU), available information (AI), quality management (QM) and trust (T) (Figure 1).

The software gave the highest value (5.25) to perceived compatibility (PC) and the lowest value (1.15) to trust (T); the rest of the factors were found above the average of these two (3.20), minus the quality management (QM) factor (3.00). These values were used to measure the degree of influence that a factor exerts on the others and were calculated from the evaluations given by the degree of causal relationships provided by the experts

**Figure 2:**
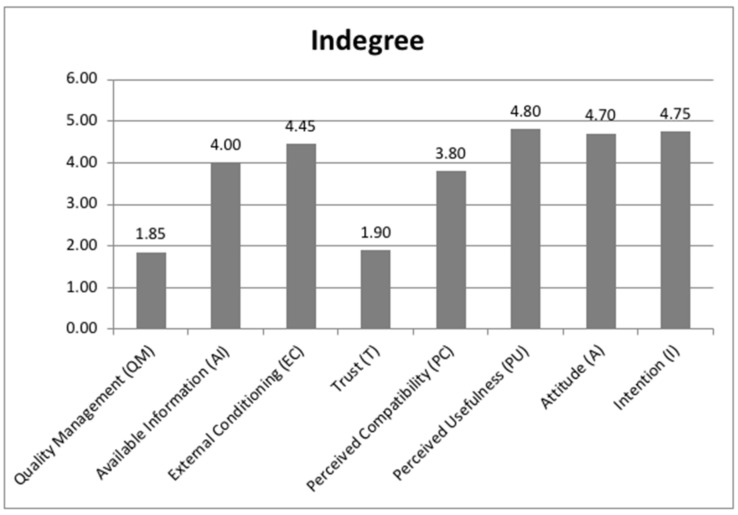
Indicators of the fuzzy cognitive map: indegree.

Regarding the indegree indicator, the classification of these factors from highest to lowest was perceived usefulness (PU), intention (I), attitude (A), external conditioning (EC), available information (AI), perceived compatibility (PC), trust (T) and quality management (QM) (Figure 2). This classification is independent of the previous classification, and its values were only given by the degree of causal relationships provided by the experts.

Its values were used to measure the degree of influences that a factor received from the other factors, and the software gave the highest value (4.80) to perceived usefulness (PU) and the lowest value (1.85) to quality management (QM); the rest were found above the mean of these two factors (3.325) minus trust (T) (1.90).

**Figure 3:**
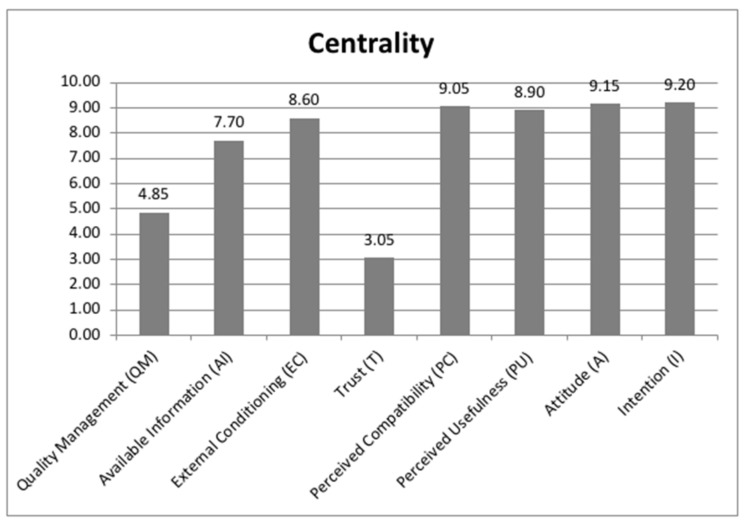
Indicators of the fuzzy cognitive map: centrality.

Regarding the centrality indicator, the classification of these factors from highest to lowest was intention (I), attitude (A), perceived compatibility (PC), perceived usefulness (PU), external conditioning (EC), available information (AI), quality management (QM) and trust (T) (Figure 3). This classification is dependent on the previous classifications because it is the result of the sum of the outdegree and indegree values for each factor.

The software gave the highest value (9.20) to intention (I) and the lowest value (3.05) to trust (T); the rest of the factors were above the mean of these two factors (6.125) minus quality management (QM) (4.85).

## 5. Discussion and Conclusions

The use of technological tools was key to ensuring that the education sector did not suffer significantly from the mobility restrictions imposed by the Spanish government during the COVID-19 pandemic. This led to effective virtual teaching and the use of tools that many teachers had never before thought of incorporating into their teaching and training and which could continue to be used after a return to traditional training.

Among these tools is videoconferencing and its use in university tutorials. In this way, this study tried to locate the key motivational factors for teachers to use it to complement face-to-face tutorials by determining what were the most influential or decisive factors.

In this study, the degree of influence of each factor was not assessed independently; rather, it took into account how factors influence each other, which might change a factor’s relevance in making a decision. This fact had not been taken into account in previous studies.

To do this, a list of the most influential motivational factors was presented. The list consisted of quality management (QM), available information (AI), external conditioning (EC), trust (T), perceived compatibility (PC), perceived usefulness (PU), attitude (A) and intention (I).

The role of all these factors in this decision was confirmed, and in order of relevance, they were intention (I), attitude (A), perceived compatibility (PC), perceived usefulness (PU), external conditioning (EC), available information (AI), quality management (QM) and trust (T).

It was observed that the factors that exerted the most influence were those that affected the character of the teaching staff toward videoconferencing (perceived compatibility, attitude and intention). On the other hand, trust and quality management had the least influence on the other factors because the safety and quality of these tools were not considered important.

These less-influential factors coincided with the factors reached in another study on motivational factors concerning the inclusion of digital tools in teaching [88] but were different for e-proctoring [43] where trust was the most influential factor, and cloud computing [89] where quality management was the second most influential. In teaching, the key factors for inclusion were intention, attitude and perceived compatibility; for cloud computing, they were external conditioning and quality management [89]; and for e-proctoring, they were trust and attitude [43]. For this reason, a study of motivational factors must be carried out for each tool to be included in teaching.

With all this, this study answered the question of what were the most influential factors for teachers to decide to use videoconferencing. These were intention, attitude and perceived compatibility; factors such as usefulness, external conditioning or pressures, available information on its use, quality management and trust were not deemed to be of much relevance.

## Figures and Tables

**Table 1:** Motivational factors: component concepts of the fuzzy cognitive map.

Variable	Definition
Quality Management	The degree of satisfaction when using videoconferences as a means in which to carry out tutorials in universities in the post-pandemic period as a complement to face-to-face tutorials, from the point of view of the perceived quality of use and the measurable results obtained.
Available Information	The degree of adoption and use of videoconferencing as a means in which to carry out tutorials in universities in the post-pandemic period as a complement to face-to-face tutorials based on existing information.
External Conditioning	The influence of the environment on the potential users of videoconferences as a means in which to carry out tutorials in universities in the post-pandemic period as a complement to face-to-face tutoring, whether favorable or unfavorable.
Trust	The degree of security and privacy expected by users regarding the use of videoconferences as a means in which to carry out tutorials in universities in the post-pandemic period as a complement to face-to-face tutorials.
Perceived Compatibility	The degree of compatibility perceived by the user regarding the use of videoconferences as a means in which to carry out tutorials in universities in the post-pandemic period as a complement to face-to-face tutorials.
Perceived Usefulness	The increase in the user’s own performance with the use of videoconferences as a means in which to carry out tutorials in universities in the post-pandemic period as a complement to face-to-face tutorials.
Attitude	The perception (in the user) of the advantage or disadvantage obtained in the behavior carried out when using videoconferences as a means in which to carry out tutorials in universities in the post-pandemic period as a complement to face-to-face tutorials.
Intention	Motivational elements that influence the use or not of videoconferences as a means in which to carry out tutorials in universities in the post-pandemic period as a complement to face-to-face tutorials.

**Table 2:** Numerical assessment of cause–effect relationships.

Degrees of Causal Relationships	Numerical Equivalence
Very strongly positive	1
	0.9
Strongly positive	0.8
	0.7
Medium positive	0.6
	0.5
Weakly positive	0.4
	0.3
Very weakly positive	0.2
	0.1
There is no relationship	0
	−0.1
Very weakly negative	−0.2
	−0.3
Weakly negative	−0.4
	−0.5
Medium negative	−0.6
	−0.7
Strongly negative	−0.8
	−0.9
Very strongly negative	−1

**Table 3:** Adjacent matrix of the collective fuzzy cognitive map.

	Quality Management (QM)	Available Information (AI)	External Conditioning (EC)	Trust (T)	Perceived Compatibility (PC)	Perceived Usefulness (PU)	Attitude (A)	Intention (I)
**Quality Management (QM)**	-	0.60	0.80	0.00	0.20	0.60	0.40	0.40
**Available Information (AI)**	0.30	-	0.50	0.40	0.70	0.60	0.60	0.60
**External Conditioning (EC)**	0.00	0.40	-	0.60	0.75	0.80	0.80	0.80
**Trust (T)**	0.10	0.25	0.20	-	0.10	0.10	0.20	0.20
**Perceived Compatibility (PC)**	0.70	0.80	0.90	0.15	-	0.90	0.90	0.90
**Perceived Usefulness (PU)**	0.10	0.80	0.85	0.10	0.45	-	0.90	0.90
**Attitude (A)**	0.35	0.60	0.70	0.15	0.80	0.90	-	0.95
**Intention (I)**	0.30	0.55	0.50	0.50	0.80	0.90	0.90	-

**Table 4:** Factors with higher centrality, outdegree and indegree indicators.

Outdegree	Indegree	Centrality
Perceived Compatibility (PC)	Perceived Usefulness (PU)	Intention (I)
Intention (I)	Intention (I)	Attitude (A)
Attitude (A)	Attitude (A)	Perceived Compatibility (PC)

## Data Availability

Not applicable.
